# Challenges in accurate HDV RNA quantification: inter-assay variability and the impact of thermal shock

**DOI:** 10.1128/jcm.01517-25

**Published:** 2025-12-31

**Authors:** Felipe Pérez-García, Ana Virseda-Berdices, Carlos Pita-Martínez, Mario Muñoz Monte, Daniel Sepúlveda-Crespo, Helena Codina, Roberto Alonso, Lara Mesones, Sandra Rodrigo, Juan Macías, Luis Miguel Real, Juan Cuadros-González, Isidoro Martínez, Salvador Resino

**Affiliations:** 1 Servicio de Microbiología Clínica, Hospital Universitario Príncipe de Asturias 16269 , Madrid, Spain; 2 Universidad de Alcalá, Facultad de Medicina, Departamento de Biomedicina y Biotecnología 16720, Madrid, Spain; 3 Centro de Investigación Biomédica en Red en Enfermedades Infecciosas (CIBERINFEC), Instituto de Salud Carlos III 38176, Madrid, Spain; 4 Unidad de Infección Viral e Inmunidad, Centro Nacional de Microbiología, Instituto de Salud Carlos III, Madrid, Spain; 5 Servicio de Microbiología Clínica y Enfermedades Infecciosas, Hospital General Universitario Gregorio Marañón 16483, Madrid, Spain; 6 Instituto de Investigación Sanitaria Gregorio Marañón 559924 , Madrid, Spain; 7 Universidad Complutense de Madrid, Facultad de Medicina 16734, Madrid, Spain; 8 CIBER Enfermedades Respiratorias-CIBERES, Madrid, Spain; 9 Hospital Universitario Virgen de Valme, Sevilla, Spain; 10 Instituto de Biomedicina de Sevilla (IBiS), Sevilla, Spain; 11 Departamento de Medicina, Universidad de Sevilla 16778, Sevilla, Spain; 12 Departamento de Bioquímica Médica, Biología Molecular e Inmunología, Universidad de Sevilla 16778, Sevilla, Spain; Mayo Clinic Minnesota, Rochester, Minnesota

**Keywords:** hepatitis D virus, qRT-PCR, inter-assay variability, viral load, thermal shock

## Abstract

Quantitative RT-PCR (qRT-PCR) is essential for monitoring hepatitis delta virus (HDV) RNA, yet assays lack standardization. We aimed to evaluate the performance of three qRT-PCR assays and to assess the impact of a pre-analytical thermal shock procedure. We conducted a comparative study using 206 samples (106 with anti-HDV antibodies and 100 anti-HDV-negative as a control group), which were tested in parallel with three qRT-PCR assays: Vircell, Certest, and Altona. Performance was evaluated for inter-assay agreement (kappa index), quantitative correlation (R²), and bias (Bland-Altman). Altona detected 56 HDV-RNA positive samples, whereas Vircell and Certest detected 55 positive samples. Inter-assay agreement was perfect comparing Vircell vs Certest (agreement = 100%, к = 1.000) and almost perfect comparing Altona with both Vircell and Certest (agreement = 99.5%, к = 0.988). Quantitatively, Vircell and Certest assays showed relevant systematic biases compared to Altona, overestimating viral loads by approximately 0.24 log_10_ International Units (IU)/mL (Certest) and 0.33 log_10_ IU/mL (Vircell). The correlation with Altona was strong for Certest (R² = 0.864) and moderate for Vircell (R² = 0.793), while the correlation between Certest and Vircell was weaker (R² = 0.720). Thermal shock improved sensitivity in one case (Certest vs Vircell) but increased quantitative variability, worsening the inter-assay correlation (R² = 0.684). In conclusion, all three assays were highly concordant for the qualitative diagnosis of HDV infection, but their quantitative biases prevent their interchangeable use for treatment monitoring. Thermal shock is not recommended for routine monitoring, as a significant compromise in quantitative accuracy and precision outweighs any potential gains in sensitivity.

**IMPORTANCE:**

This study evaluates three hepatitis delta virus (HDV) RNA quantitative real-time PCR (qRT-PCR) assays, crucial for managing the HDV infection, particularly in the setting of new therapies like Bulevirtide, where assessing viral load reduction and accurate monitoring is paramount. We reveal significant quantitative biases among widely used assays, precluding their interchangeable use and risking misinterpretation of treatment response. Furthermore, our systematic assessment of the thermal shock pre-analytical procedure highlights its detrimental impact on quantitative precision, despite modest sensitivity gains. This work provides essential evidence for clinicians and laboratories, guiding assay selection and standardization efforts to optimize HDV diagnosis and patient monitoring.

## INTRODUCTION

Hepatitis D virus (HDV), a unique RNA pathogen dependent on the hepatitis B surface antigen (HBsAg), causes a highly aggressive form of viral hepatitis, rapidly accelerating progression to cirrhosis and hepatocellular carcinoma (1). Despite its clinical severity, the global burden of HDV is poorly understood, with prevalence estimates differing dramatically from 12 million to over 70 million people worldwide (1–4). This highlights the critical role of reliable diagnostic assays in both clinical management and accurate epidemiological surveillance.

Adding to the challenge of managing this global pathogen is its considerable genetic diversity. HDV is classified into eight distinct genotypes, each with a specific geographical distribution: genotype 1 is ubiquitous, while others are more restricted, such as genotype 3 in South America and genotypes 5–7 in parts of Africa (1, 5). This genetic heterogeneity, coupled with the virus’s highly stable, rod-like secondary RNA structure, poses a significant challenge for molecular diagnostic assays, as primers and probes must reliably detect and quantify all circulating variants to ensure accurate diagnosis and effective treatment monitoring (6). Failure to account for this complexity can lead to false-negative results or inaccurate viral load measurements, directly compromising patient care (7). This is particularly relevant in an interconnected world where diverse genotypes may be encountered outside their traditional endemic regions.

The approval of Bulevirtide, a first-in-class entry inhibitor, has revolutionized the treatment landscape for chronic HDV (8, 9). This marks a significant advance over previous therapies like pegylated interferon alpha, which offered low rates of sustained virologic response (25%–30%) (9, 10). The efficacy of Bulevirtide is monitored by measuring a significant reduction in HDV RNA, typically defined as a decline of ≥2 log_10_ International Units (IU)/mL or achieving undetectable RNA levels (8–11). This new therapeutic paradigm has shifted quantitative real-time PCR (qRT-PCR) from a simple diagnostic tool to an essential component of clinical decision-making, making the accuracy and reliability of viral load measurements more critical than ever. In this context, even modest, systematic biases between assays—such as those of 0.3 to 0.5 log_10_ IU/mL—could lead to the misinterpretation of virologic response, potentially resulting in premature discontinuation or unnecessary continuation of therapy.

Additionally, a notable lack of standardization, with no Food and Drug Administration-approved assays and only a limited number of Conformité Européenne (CE)-marked *in vitro* diagnostics (6), has led to significant inter-assay variability (12). Consequently, many clinical laboratories rely on research-use-only (RUO) kits, whose performance can vary. This challenge is further complicated by pre-analytical procedures, such as the debated “thermal shock” procedure, a heating step proposed to improve RNA denaturation and quantification, by disrupting HDV RNA secondary structure before amplification (7, 13). The lack of consensus on both the choice of assay and optimal pre-analytical protocols creates a significant risk of inconsistent results, directly impacting patient management.

This diagnostic gap underscores an urgent need for head-to-head comparisons of available assays and clarification on the utility of pre-analytical steps like thermal shock. Therefore, this study aimed to compare the agreement and quantitative performance of three RUO qRT-PCR assays (VIASURE HDV q Real-Time PCR Detection Kit [Certest]; Hepatitis Delta Real-time PCR kit [Vircell]; and AltoStar HDV PCR Kit 1.5 [Altona]). Concurrently, we systematically evaluated the impact of a pre-analytical thermal shock procedure on HDV RNA quantification.

## MATERIALS AND METHODS

### Study design and samples

This cross-sectional single-center study included 206 participants with a cryopreserved at −80°C serum (*n* = 150) or plasma (*n* = 56) sample collected between July 2021 and September 2024. We divided samples into two groups: (i) an HDV-positive group (*n* = 106) consisting of HBsAg-positive and anti-HDV-positive patients. Among these, 56 (52.8%) were coinfected with HIV; (ii) a control group (*n* = 100) comprising 50 HBsAg-negative individuals and 50 HBsAg-positive/anti-HDV-negative individuals. The samples were obtained from three Spanish hospitals: Hospital Universitario Príncipe de Asturias, Hospital General Universitario Gregorio Marañón, and Hospital Universitario Virgen de Valme. All laboratory procedures were performed between September and November 2024 at Hospital Universitario Príncipe de Asturias. To minimize pre-analytical variability, all aliquots used for this study were subjected to a single freeze-thaw cycle.

The study was approved by the Ethics Committee of the Hospital Universitario Príncipe de Asturias (Ref: LIB 18/2023). It adhered to the ethical principles of the Declaration of Helsinki and was conducted according to the Standards for Reporting Diagnostic Accuracy (STARD) guidelines (14) (Table S1).

### Laboratory assays

#### Serologic assays

HBV and HDV serological tests were performed using two assays based on chemiluminescence immunoassay technology. HBsAg was detected with the HBsII assay (Siemens Healthineers, Erlangen, Germany), and anti-HDV antibodies were detected with the Liaison XL murex Anti-HDV assay (DiaSorin, Saluggia, Italy).

#### Molecular assays

Nucleic acids were extracted from 400 µL of each sample using the MagCore HF16 system (Werfen, Barcelona, Spain) with a no. 203 high-sensitivity cartridge. The final elution volume was 40 µL.

qRT-PCR was performed on a CFX Opus 96 Real-Time PCR System (Bio-Rad, California, USA). We compared the following three RUO assays: (i) VIASURE HDV q Real-Time PCR Detection Kit (Certest, Zaragoza, Spain); (ii) Hepatitis Delta Real-time PCR kit (Vircell, Granada, Spain); (iii) AltoStar HDV PCR Kit 1.5 (Altona Diagnostics, Hamburg, Germany). For performance details of each assay, see Table 1. All assays employed quantification standards that were calibrated against the first WHO International Standard for Hepatitis D Virus RNA for Nucleic Acid Amplification Techniques-based assays (PEI code: 7657/12).

**TABLE 1:** Performance details for each qRT-PCR assay evaluated in this study^*a*^

	VIASURE *HDV q* real-time PCR detection kit (Certest)	Hepatitis Delta real-time PCR kit (Vircell)	AltoStar HDV PCR kit (Altona)
Target genomic region	*ribozyme* region	*HDAg-L* gene	Not specified in the IFU
Limit of detection	157.85 IU/mL (EDTA-plasma) 68.36 IU/mL (serum)	23 IU/mL	1.12 IU/mL
Range of quantification	2.5 × 10^2^–2.5 × 10^5^ IU/mL (EDTA-plasma) 6.9 × 10^2^–6.9 × 10^5^ IU/mL (serum)	1.0 × 10^4^–1.0 × 10^7^ IU/mL	1.0 × 10^4^–1.0 × 10^7^ IU/mL
Internal control	Endogenous internal control to monitor sample extraction and PCR reaction	Exogenous internal control to monitor PCR reaction	Exogenous internal control to monitor PCR reaction
HDV genotypes detected	1–8	1–8	1–8
Sample input volume (µL)	5	5	45
Validated sample matrices	Human EDTA plasma and serum	Human EDTA plasma and serum	Human EDTA plasma and serum
Reagent format	Lyophilized	Lyophilized	Liquid
Storage conditions	Room temperature	2°C to 8°C	−25°C to −15°C (avoid repeated freeze/thaw)
Regulatory status	RUO	RUO	RUO

^*a*^ qRT-PCR, quantitative real-time polymerase chain reaction; RUO, research-use-only; IU, International Units; IFU, instructions for use; HDV, hepatitis D virus; HDAg, hepatitis D antigen.

All assays were evaluated against each other, and all procedures followed the manufacturer’s instructions.

#### Thermal shock procedure

In parallel with the standard workflow, we applied a thermal shock procedure to extracted RNA aliquots to assess its potential to enhance HDV RNA quantification by disrupting its secondary structure before amplification (7, 13, 15). The RNA eluates were incubated at 95°C for 10 min and then immediately cooled at −20°C for at least 10 min.

#### HDV genotyping

HDV-RNA positive samples were genotyped by conventional PCR and Sanger sequencing. A 369 bp fragment of the RT coding region was amplified using the primers F: 5′-CATGCCGACCCGAAGAGGAAAG-3′ and R: 5′-GAAGGAAGGCCCTCGAGAACAAGA-3′.

PCR was performed in 20 µL reactions containing 1 µL of HDV RNA and 0.2 µL of Ex Taq HS (TaKaRa Bio, Tokyo, Japan). Thermal cycling conditions were an initial denaturation at 95°C for 5 min; 40 cycles of 95°C for 45 s, 58°C for 45 s, and 72°C for 30 s; and a final extension at 72°C for 5 min. PCR products were visualized by agarose gel electrophoresis.

Amplicons were then purified and sequenced. The resulting sequences were manually assembled and edited using CodonCode Aligner software (CodonCode Corp., Dedham, MA). Genotypes were identified using the NCBI BLAST tool.

### Statistical analysis

Continuous variables are presented as median and interquartile range (IQR), while categorical variables are presented as counts and proportions. Group comparisons were performed using the Mann-Whitney U test for continuous data. A two-tailed *P* value <0.05 was considered statistically significant.

Agreement between assays was assessed using Cohen’s kappa coefficient (κ). The correlation of quantitative HDV RNA results (expressed as log_10_ IU/mL) between assays was analyzed using Pearson’s correlation coefficient (R²). Bland-Altman plots were used to assess systematic bias and the limits of agreement. For quantitative analyses (Pearson and Bland-Altman), only samples with viral loads within the quantification range of the assays were included.

All statistical analyses were performed using Stata version 19.5 (StataCorp LLC, College Station, TX, USA). Figures were generated with GraphPad version 10.3.1 (GraphPad Software, Inc., San Diego, CA, USA).

## RESULTS

### Qualitative agreement results

All control group samples (*n* = 100) were negative across the three qRT-PCR assays. Among the 106 samples from the HDV-positive group, the Altona assay detected HDV RNA in 56 (52.8%), whereas the Vircell and Certest assays identified 55 (51.9%). The single discordance occurred in a sample with a very low viral load (4.87 IU/mL, as quantified by Altona). Vircell assay reported this sample as negative, while Certest assay yielded a cycle threshold (Ct) value of 40.37, which was just above the assay’s positivity cutoff (Ct ≤ 40.00). Of the 56 HDV RNA-positive samples, genotyping was successful for 41, all of which were identified as HDV genotype 1, with one sample further subtyped as 1d.

Vircell and Certest assays demonstrated perfect qualitative agreement (100% concordance, κ = 1.000). When compared to the Altona assay, both Vircell and Certest showed near-perfect agreement, with an overall concordance of 99.5% and a Kappa score of 0.988.

### Quantitative results

**Fig 1:**
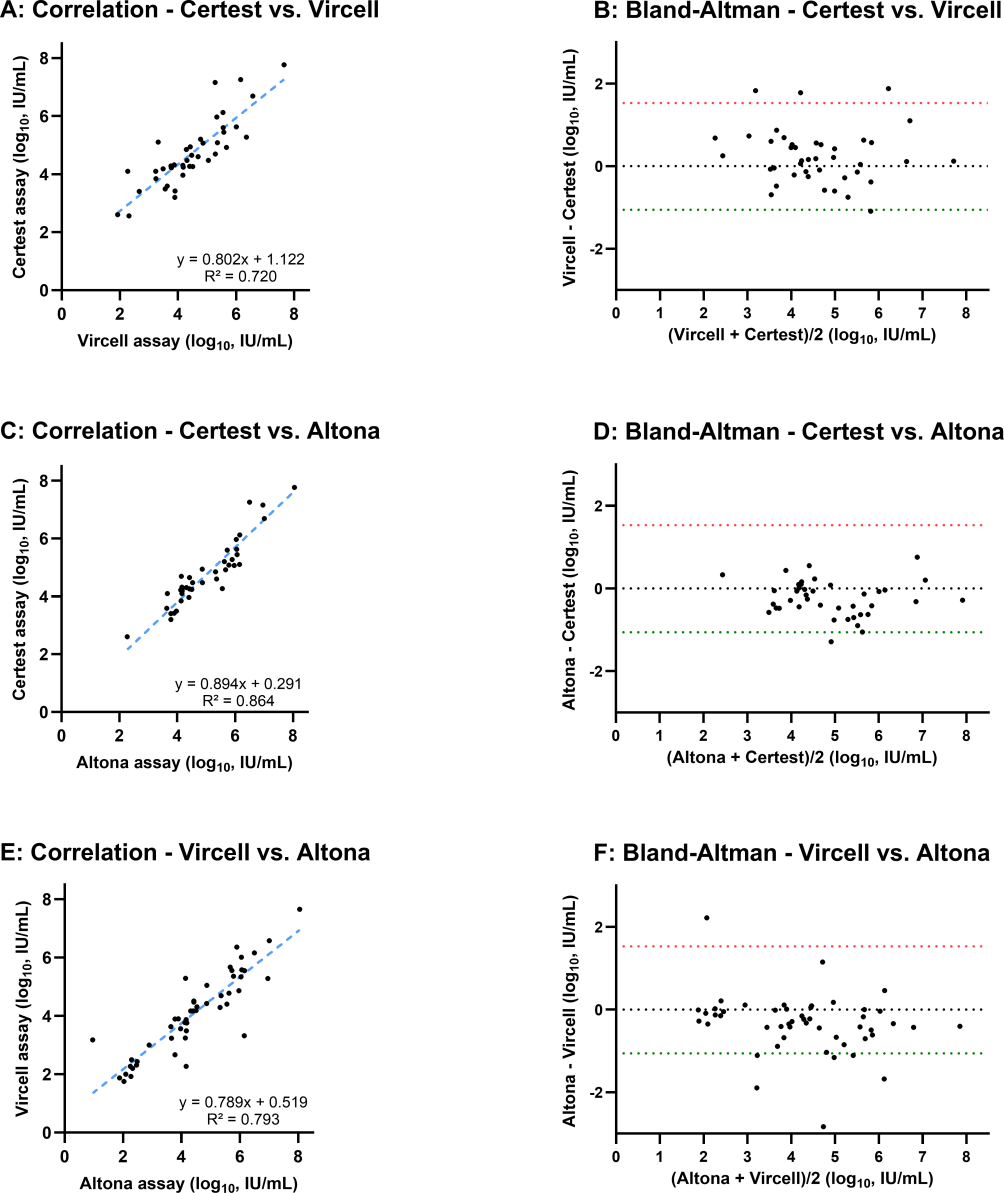
Correlation and Bland-Altman analyses between quantitative HDV RNA assays. The figure presents a pairwise comparison of viral load measurements (log_10_ IU/mL) from the three qRT-PCR assays. Panels **A, C, and E** are scatter plots showing the correlation between assays. The dashed blue line represents the linear regression, with the corresponding equation and coefficient of determination (R²) displayed for each comparison. Panels** B, D, and F** are Bland-Altman plots assessing the systematic bias. The Y-axis shows the difference between the two assays, while the X-axis shows their mean value. The dotted black line indicates the mean difference (bias), with a value near zero suggesting a lack of systematic disagreement between the assays. The dashed red and green lines represent the 95% limits of agreement (mean ± 1.96 SD). The specific comparisons shown are (**A and B**) Certest vs Vircell; (**C and D**) Certest vs Altona; and (**E and F**) Vircell vs Altona. HDV, hepatitis D virus; IU, International Unit; R², coefficient of determination; RNA, ribonucleic acid; SD, standard deviation.

For quantitative analysis, results were available for 51 of the 56 HDV RNA-positive samples, as five samples were below the range of quantification for all assays. The correlation and agreement between the assays are detailed in Fig. 1. Ten additional samples for Certest and two for Vircell were excluded from pairwise quantitative comparisons as their viral loads were outside the linear range of the respective assay.

The comparison between Certest and Vircell assays yielded the weakest correlation (R² = 0.720; Fig. 1A). The corresponding Bland-Altman analysis revealed a small positive mean bias of 0.238 log_10_ IU/mL (Vircell–Certest), indicating that Vircell tended to report slightly higher viral loads than Certest (Fig. 1B). Despite this bias, 90.2% of values fell within the limits of agreement.

In contrast, the correlation between Certest and Altona assays was the strongest (R² = 0.864; Fig. 1C). However, the Bland-Altman plot identified a relevant systematic negative bias of −0.239 log_10_ IU/mL (Altona–Certest), indicating that Certest assay consistently reported higher viral loads than the Altona assay (Fig. 1D). Similarly, the comparison between Vircell and Altona yielded a moderate correlation (R² = 0.793; Fig. 1E) and revealed a larger systematic negative bias of −0.334 log_10_ IU/mL (Altona–Vircell), confirming that the Vircell assay also yielded higher viral loads than Altona (Fig. 1F). When comparing both Certest and Vircell to Altona, 92.2% of the measurements fell within the limits of agreement.

### Thermal shock results

**Fig 2:**
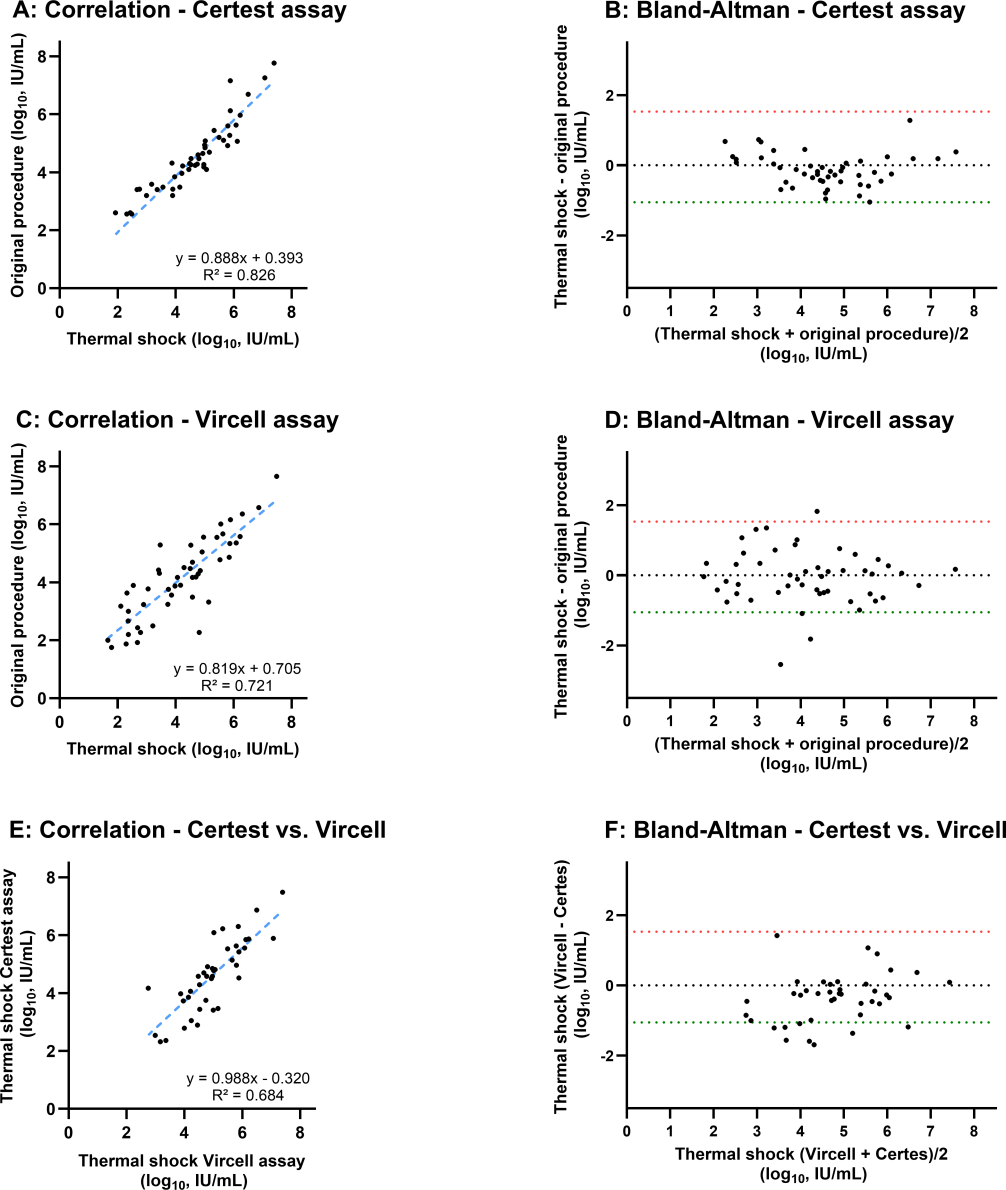
Impact of a pre-analytical thermal shock procedure on HDV RNA quantification and inter-assay agreement. This figure evaluates the intra-assay variability and inter-assay agreement after applying the thermal shock procedure. Panels** A, C, and E** are scatter plots showing correlations. The dashed blue line represents the linear regression, with the corresponding equation and coefficient of determination (R²) displayed for each comparison. Panels** B, D, and F** are Bland-Altman plots assessing systematic bias. The Y-axis shows the difference between measurements, while the X-axis shows their mean value. The dotted black line indicates the mean difference (bias), with a value near zero suggesting a lack of systematic disagreement between the assays. The dashed red and green lines represent the 95% limits of agreement (mean ± 1.96 SD). The specific comparisons shown are (**A and B**) intra-assay analysis for Certest: compares results from the standard vs thermal shock procedures. (**C and D**) Intra-assay analysis for Vircell: compares results from the standard vs thermal shock procedures. (**E and F**) Inter-assay analysis post-thermal shock: compares results from Certest vs Vircell after applying the thermal shock.

The pre-analytical thermal shock procedure was performed on all 56 HDV RNA-positive samples using the Vircell and Certest assays; insufficient sample volume precluded its assessment with the Altona assay. The main results are summarized in Table 2 and Fig. 2.

**TABLE 2:** Comparison of HDV RNA quantification metrics with and without a pre-analytical thermal shock procedure^*a*^^,*b*^

		Thermal shock procedure		
Variable	qRT-PCR assay	Yes	No	*P* value
Ct value	Vircell	26.99 (23.05–30.75)	26.89 (23.36–30.28)	0.658
	Certest	26.56 (22.56–34.42)	26.50 (22.78–36.10)	0.765
Viral load (log_10_ IU/mL)	Vircell	4.17 (2.89–5.53)	4.17 (3.23–5.34)	0.697
	Certest	4.53 (2.41–5.53)	4.25 (2.22–5.22)	0.700

^*a*^ Statistics: all values are presented as median (IQR). The *P* values represent the statistical comparison between the two conditions (with vs without thermal shock) for each variable.

^*b*^ Ct, cycle threshold; HDV, hepatitis D virus; IQR, interquartile range; qRT-PCR, quantitative real-time polymerase chain reaction.

Overall, the thermal shock procedure did not lead to statistically significant changes in the median Ct values or median viral loads for either assay (all *P* > 0.65, Table 2). However, intra-assay analysis revealed that the procedure introduced considerable quantitative variability. Although the correlations between the standard and thermal shock procedures were strong for Certest (R² = 0.826; Fig. 2A) and moderate for Vircell (R² = 0.721; Fig. 2C), the Bland-Altman plots demonstrated wide limits of agreement (Certest [bias = −0.0508; Fig. 2B] and Vircell [bias = −0.0508; Fig. 2D]), indicating inconsistent effects on individual samples. Additionally, the Bland-Altman plot comparing the Vircell and Certest assays post-thermal shock (Fig. 2F) showed a negligible mean bias (−0.0508 log_10_ IU/mL). However, the analysis revealed wide limits of agreement, indicating that the procedure introduced considerable random variability and resulted in poor quantitative concordance between the two assays.

Crucially, this added variability impaired the quantitative agreement between the two assays. The correlation coefficient (R²) between Certest and Vircell decreased from 0.720 with the standard procedure (Fig. 1A) to 0.684 post-thermal shock (Fig. 2E). Furthermore, the post-thermal shock Bland-Altman analysis (Fig. 2F) confirmed an insignificant mean bias (−0.0508 log_10_ IU/mL) but revealed wide limits of agreement, demonstrating poor quantitative concordance between the assays.

Thus, the stable median values masked a significant and heterogeneous impact on individual samples. The procedure successfully achieved HDV RNA detection in the single discordant sample in both assays (Vircell Ct = 38.16; Certest Ct = 38.80). Moreover, it induced a substantial viral load increase (>0.5 log_10_ IU/mL) in a notable subset of samples (19.1% for Certest and 24.5% for Vircell). These findings highlight the procedure’s capacity to improve detection in low-viremia cases but also underscore the unpredictable variability it introduces, compromising its utility for reliable quantification.

## DISCUSSION

This study confirms that while Vircell, Certest, and Altona HDV RNA assays are reliable for initial diagnosis, they exhibit relevant quantitative biases that preclude their interchangeable use for monitoring treatment response. Our key findings highlight an excellent qualitative concordance between assays, but systematic overestimation of viral loads depending on the comparator assay. Furthermore, we demonstrate that the pre-analytical thermal shock procedure, while potentially enhancing sensitivity in low-viremia cases, introduces unacceptable quantitative variability, compromising its utility for routine clinical use.

The high agreement of Vircell, Certest, and Altona assays is a crucial and useful finding for diagnostic laboratories. Certest and Vircell assays showed perfect agreement between them and almost perfect agreement with Altona (к = 0.988). This is consistent with recent literature reporting high concordance among different commercial assays (12). The single discrepancy observed was a sample with a very low viral load (4.87 IU/mL by Altona), which fell below the detection limit of Vircell and Certest (see Table 1). This highlights that while these assays are highly reliable for diagnosing active infection, minor differences in analytical sensitivity can affect results at the threshold of detection, a point also raised in other assay comparisons (16). This high degree of accuracy is fundamental for clinical practice, as it allows clinicians to confidently rule in or rule out active HDV infection, which is the first critical step in patient management.

While qualitatively reliable, our quantitative analysis revealed that both Certest and Vircell assays systematically overestimate HDV RNA levels compared to the Altona assay. We identified a relevant negative bias of −0.239 log_10_ IU/mL (Altona–Certest) and −0.334 log_10_ IU/mL (Altona–Vircell), indicating that Certest and Vircell report viral loads higher than Altona, respectively. This situation could be due to differences in the target HDV genomic region (see Table 1) or primer composition, which imply different amplification efficiencies. Additionally, it is important to note that in the absence of a true gold standard, Altona may underestimate viral loads. Nonetheless, this finding of systematic overestimation could be significant and aligns with other studies that underscore inter-assay variability as a major challenge in HDV management (16, 17). These discrepancies could have direct clinical implications. For patients on therapies like Bulevirtide, where virologic response is often defined by a ≥2-log_10_ decline in viral load (8, 9, 17), a systematic bias of over 0.3 log_10_ IU/mL could not be merely a statistical nuance but a quantitatively significant discrepancy. It could potentially lead to the misclassification of a patient’s response to therapy, for instance, making a non-responder appear to have achieved a partial virologic response. Given that integrated patient management depends on factors like liver function tests and clinical status, these quantitative discrepancies highlight the need for platform consistency to avoid ambiguity when defining virological milestones. In line with this, our results strongly reinforce the recommendation that sequential monitoring of a patient’s HDV RNA must be performed with the same assay to mitigate this critical inter-assay variability (12, 16, 17).

A novel aspect of our study was the systematic evaluation of a pre-analytical thermal shock. This pre-analytical step does not align with the HDV RNA package inserts recommendations. However, to enhance qRT-PCR efficiency, some authors have theorized that pre-treating the RNA eluate is a critical step. Due to its high GC content (~70%), the HDV genome forms a complex secondary structure that can hinder primer binding to the target region (18). The rationale is that a thermal shock denatures this structure, improving quantification. In line with this hypothesis, previous reports showed that this procedure could enhance sensitivity (7). Other studies using Qiagen and Vircell assays also reported improved HDV RNA quantification, with mean Ct reductions of 2.11 and 1.34, respectively, confirming enhanced sensitivity with this procedure (19). Our results, however, paint a more complex picture. We observed that it successfully converted the single discordant sample from negative to positive in both assays and induced a clinically relevant viral load increase (>0.5 log_10_ IU/mL) in a substantial subset of samples (19.1% for Certest and 24.5% for Vircell). However, this potential benefit was offset by a notable increase in quantitative variability and a decrease in inter-assay agreement. Crucially, the correlation between Certest and Vircell worsened after applying thermal shock (R² decreased from 0.720 to 0.683). This indicates that the procedure’s effect is not uniform and can amplify existing differences between assays. As these assays detect different regions of the HDV genome, thermal shock could expose some RNA regions more than others, which could explain this differential performance between assays (for example, if the Vircell assay target is located in an area with a more complex secondary structure, thermal shock could differentially affect that assay more than Certest, increasing the difference in quantification results as compared to Certest). Therefore, our data suggest that any potential benefit in sensitivity is outweighed by a critical loss in quantitative precision and a differential performance depending on the assay employed, making its routine implementation for treatment monitoring inadvisable.

### Limitations and strengths

This study has several limitations. First, our sample cohort was restricted to HDV genotype 1, which is predominant in our region. Although our findings may not be directly generalizable to other genotypes, current evidence suggests no significant variations in viral load quantification between different HDV genotypes (6), potentially mitigating the impact of this limitation. Second, due to sample volume constraints, we could not evaluate the effect of the thermal shock procedure on the Altona assay. This leaves its response to this pre-analytical step unknown, though any differential effect would only reinforce our conclusion that the procedure’s impact is assay-dependent. Third, our study lacked a gold standard comparator (e.g., a CE-marked or digital PCR assay), which would have been valuable for assessing assay reliability. However, absolute quantification using digital PCR assays could not constitute a clinical gold standard. Given the limited availability of CE-marked HDV RNA kits in Spain, comparative studies like ours are crucial for validating reliable commercial diagnostics. We could also have employed the WHO International Standard for standardizing IU/mL quantification. However, our objective was to compare the relative bias between commercially available kits in Spanish clinical practice, not their absolute calibration. Finally, the single-center design of our study precluded the assessment of inter-laboratory variability. Multi-center studies with more diverse patient populations and genotypes to assess not only inter-assay variability but also inter-laboratory variability are needed to confirm our findings and inform best practices.

Despite these limitations, our study has significant strengths. Its primary strength is the direct head-to-head comparison of three widely used assays in Spain using a robust cohort of clinical samples. Furthermore, we systematically quantified the trade-offs of a controversial pre-analytical step—thermal shock—providing much-needed data on its benefits versus its drawbacks. This comprehensive evaluation of both diagnostic accuracy and quantitative performance was conducted in strict adherence to the STARD guidelines, ensuring methodological rigor and transparent reporting.

### Conclusion

In summary, our head-to-head comparison demonstrates that Vircell, Certest, and Altona assays exhibit high reliability for the qualitative diagnosis of HDV, evidenced by excellent concordance. Conversely, the presence of relevant quantitative biases precludes their suitability for interchangeable use in treatment monitoring. To ensure accurate interpretation of viral load trends, clinicians must employ the identical assay for longitudinal patient monitoring. Moreover, the pre-analytical thermal shock procedure presents a significant limitation, as its capacity to enhance detection in certain scenarios is compromised by an unacceptable degree of quantitative variability. Consequently, its routine application for patient monitoring is not recommended.

## Data Availability

The data sets used and analyzed during the current study are available from the corresponding author upon reasonable request.

## SUPPLEMENTAL MATERIAL

Table S1 jcm.01517-25-s0001.docx Table S1: Standards for Reporting Diagnostic accuracy studies (STARD) checklist.

ASM does not own the copyrights to Supplemental Material that may be linked to, or accessed through, an article. The authors have granted ASM a non-exclusive, world-wide license to publish the Supplemental Material files. Please contact the corresponding author directly for reuse.

## References

[1] Asselah T, Rizzetto M. 2023. Hepatitis D virus infection. N Engl J Med 389:58–70. doi:10.1056/NEJMra2212151 37407002

[2] Rizzetto M, Hamid S, Negro F. 2021. The changing context of hepatitis D. J Hepatol 74:1200–1211. doi:10.1016/j.jhep.2021.01.014 33484770

[3] Stockdale AJ, Kreuels B, Henrion MYR, Giorgi E, Kyomuhangi I, de Martel C, Hutin Y, Geretti AM. 2020. The global prevalence of hepatitis D virus infection: systematic review and meta-analysis. J Hepatol 73:523–532. doi:10.1016/j.jhep.2020.04.008 32335166 PMC7438974

[4] Miao Z, Zhang S, Ou X, Li S, Ma Z, Wang W, Peppelenbosch MP, Liu J, Pan Q. 2020. Estimating the global prevalence, disease progression, and clinical outcome of hepatitis delta virus infection. J Infect Dis 221:1677–1687. doi:10.1093/infdis/jiz633 31778167 PMC7184909

[5] Le Gal F, Brichler S, Drugan T, Alloui C, Roulot D, Pawlotsky J-M, Dény P, Gordien E. 2017. Genetic diversity and worldwide distribution of the deltavirus genus: a study of 2,152 clinical strains. Hepatology 66:1826–1841. doi:10.1002/hep.29574 28992360

[6] Wedemeyer H, Leus M, Battersby TR, Glenn J, Gordien E, Kamili S, Kapoor H, Kessler HH, Lenz O, Lütgehetmann M, Mixson-Hayden T, Simon CO, Thomson M, Westman G, Miller V, Terrault N, Lampertico P, HDV RNA Assays Writing Group at the HBV Forum. 2025. HDV RNA assays: performance characteristics, clinical utility, and challenges. Hepatology 81:637–650. doi:10.1097/HEP.0000000000000584 37640384 PMC11289715

[7] Homs M, Giersch K, Blasi M, Lütgehetmann M, Buti M, Esteban R, Dandri M, Rodriguez-Frias F. 2014. Relevance of a full-length genomic RNA standard and a thermal-shock step for optimal hepatitis delta virus quantification. J Clin Microbiol 52:3334–3338. doi:10.1128/JCM.00940-14 24989607 PMC4313150

[8] Wedemeyer H, Aleman S, Brunetto MR, Blank A, Andreone P, Bogomolov P, Chulanov V, Mamonova N, Geyvandova N, Morozov V, et al.. 2023. A Phase 3, randomized trial of bulevirtide in chronic hepatitis D. N Engl J Med 389:22–32. doi:10.1056/NEJMoa2213429 37345876

[9] Wedemeyer H, Schöneweis K, Bogomolov P, Blank A, Voronkova N, Stepanova T, Sagalova O, Chulanov V, Osipenko M, Morozov V, et al.. 2023. Safety and efficacy of bulevirtide in combination with tenofovir disoproxil fumarate in patients with hepatitis B virus and hepatitis D virus coinfection (MYR202): a multicentre, randomised, parallel-group, open-label, phase 2 trial. Lancet Infect Dis 23:117–129. doi:10.1016/S1473-3099(22)00318-8 36113537

[10] Asselah T, Chulanov V, Lampertico P, Wedemeyer H, Streinu-Cercel A, Pântea V, Lazar S, Placinta G, Gherlan GS, Bogomolov P, et al.. 2024. Bulevirtide combined with pegylated interferon for chronic hepatitis D. N Engl J Med 391:133–143. doi:10.1056/NEJMoa2314134 38842520

[11] Yurdaydin C, Abbas Z, Buti M, Cornberg M, Esteban R, Etzion O, Gane EJ, Gish RG, Glenn JS, Hamid S, et al.. 2019. Treating chronic hepatitis delta: the need for surrogate markers of treatment efficacy. J Hepatol 70:1008–1015. doi:10.1016/j.jhep.2018.12.022 30982526

[12] Illescas-López M, Chaves-Blanco L, de Salazar A, Hernández-Febles M, Carracedo R, Lagarejos E, Fuentes A, Pereira S, Cea M, De La Iglesia A, Freyre C, Iborra A, Odero V, García-Barrionuevo A, Aguilera A, Pena MJ, García F. 2024. Assessment of performance and comparison of three commercial HDV RNA detection assays: implications for diagnosis and treatment monitoring. Front Cell Infect Microbiol 14:1422299. doi:10.3389/fcimb.2024.1422299 38988808 PMC11233439

[13] Tian Y, Fan Z, Zhang X, Xu L, Cao Y, Pan Z, Mo Y, Gao Y, Zheng S, Huang J, Zou H, Duan Z, Li H, Ren F. 2023. CRISPR/Cas13a-assisted accurate and portable hepatitis D virus RNA detection. Emerg Microbes Infect 12:2276337. doi:10.1080/22221751.2023.2276337 37882492 PMC10796118

[14] Bossuyt PM, Reitsma JB, Bruns DE, Gatsonis CA, Glasziou PP, Irwig L, Lijmer JG, Moher D, Rennie D, de Vet HCW, Kressel HY, Rifai N, Golub RM, Altman DG, Hooft L, Korevaar DA, Cohen JF, STARD Group. 2015. STARD 2015: an updated list of essential items for reporting diagnostic accuracy studies. BMJ 351:h5527. doi:10.1136/bmj.h5527 26511519 PMC4623764

[15] Lempp FA, Schlund F, Rieble L, Nussbaum L, Link C, Zhang Z, Ni Y, Urban S. 2019. Recapitulation of HDV infection in a fully permissive hepatoma cell line allows efficient drug evaluation. Nat Commun 10:2265. doi:10.1038/s41467-019-10211-2 31118422 PMC6531471

[16] Zulian V, Taibi C, Coppola A, Bibbò A, Federici L, De Sanctis M, Pauciullo S, D’Offizi G, Biliotti E, McPhee F, Garbuglia AR. 2025. Improving virological monitoring of HDV infection: a proof-of-concept comparative study of bosphore and altostar assays in patients treated with bulevirtide. Biomedicines 13:1564. doi:10.3390/biomedicines13071564 40722639 PMC12292241

[17] Anolli MP, Uceda Renteria S, Degasperi E, Facchetti F, Sambarino D, Borghi M, Perbellini R, Soffredini R, Monico S, Callegaro A, Lampertico P. 2025. Comparing methods for plasma HDV RNA quantification in bulevirtide-treated and untreated patients with HDV. JHEP Rep 7:101299. doi:10.1016/j.jhepr.2024.101299 40051411 PMC11883403

[18] Pflüger LS, Volz T, Giersch K, Allweiss L, Dandri M, Lütgehetmann M. 2024. Method for quantitative detection: i, manual workflow HDV RNA Detection: I, Manual Workflow (Serum and Liver Tissue) and II, fully automated high throughput workflow for diagnostic use. Methods Mol Biol 2837:171–184. doi:10.1007/978-1-0716-4027-2_15 39044084

[19] Vendrell Bernal R, Iglesies Torrent J, Sellarès Crous A, Forns X, Lens S, Pérez Del Pulgar S, Leonel T, Hurtado JC. 2024. Evaluación del rendimiento de una PCR comercial para la detección de ARN de VHD comparada con una PCR casera. Zaragoza Abstr XXVII Congreso Nacional de la Sociedad Española de Enfermedades Infecciosas y Microbiología Clínica

